# Impact of digital surgery scheduling systems on the quality of preoperative care: a systematic review protocol

**DOI:** 10.1136/bmjopen-2025-102034

**Published:** 2025-07-16

**Authors:** Elena Lammila-Escalera, Gabriele Kerr, Geva Greenfield, Benedict Hayhoe, Natalie Brewer, Grazia Antonacci, Azeem Majeed, Ana Luisa Neves

**Affiliations:** 1 Department of Primary Care and Public Health, Imperial College London, London, UK; 2 Chelsea and Westminster Hospital NHS Foundation Trust, London, UK

**Keywords:** Health informatics, Quality in health care, HEALTH SERVICES ADMINISTRATION & MANAGEMENT, SURGERY

## Abstract

**Abstract:**

**Introduction:**

Ineffective surgery scheduling fails to align demand with need, resulting in financial waste, resource inefficiencies and delays in care, which ultimately lead to poorer patient outcomes. Digital systems present a promising approach to optimising scheduling. However, research examining their impact remains limited. This planned systematic review aims to evaluate the effects of digital surgery scheduling systems on the quality of preoperative care.

**Methods and analysis:**

A systematic review will be undertaken using Ovid MEDLINE, Ovid EMBASE, HMIC and PsycINFO (from inception to the present). The outcomes under investigation include the domains of quality of care (eg, patient-centredness, safety, effectiveness, efficiency, timeliness of care and equity). Two independent reviewers will screen and extract data, resolving any disagreements through discussion. Once eligible studies are identified, the extracted data will be summarised in a table. The risk of bias in the articles will be evaluated using the appropriate National Heart, Lung and Blood Institute quality qssessment tool, depending on the study design. A subgroup analysis will be carried out using demographic variables supported by the data. A narrative synthesis and a meta-analysis will be performed, to quantify the impact of digital surgery scheduling tools on reported outcomes.

**Ethics and dissemination:**

This proposed review aims to collate and summarise peer-reviewed, published evidence, and therefore, does not require ethical approval. This protocol and the subsequent review will be disseminated in peer-reviewed journals, at conferences and through patient-led lay summaries. PROSPERO registration number: CRD42024625469.

**STRENGTHS AND LIMITATIONS OF THIS STUDY**

- This systematic review will be the first to assess the practical implementation of digital surgery scheduling systems, providing actionable insights for hospital decision-makers.
- It will address urgent post-pandemic healthcare challenges by examining how these tools may optimise efficiency in scheduling.
- As surgical scheduling is multifaceted, heterogeneity is expected, which may complicate the task of isolating the direct impact of these scheduling systems.

## Introduction

 Globally, the rising demand for hospital services, driven by demographic changes such as ageing populations and an increasing prevalence of chronic diseases, places significant pressure on healthcare systems.^1^ In the UK, hospital care constitutes over 50% of NHS (National Health Service) expenditure, with operating theatres being particularly resource-intensive.^2 3^ Inefficient use of operating theatres is expensive, with lost time estimated to cost the NHS £400 million annually.^4^ These inefficiencies are often linked to poor surgical scheduling, which must balance the needs of elective and emergency procedures while ensuring the effective use of available resources.

Surgery scheduling must also balance efficiency with responsiveness to patient needs while prioritising patient safety. There are three categories of scheduling and capacity decision-making: strategic, tactical and operational. Strategic decisions, such as case-mix planning, are made a year in advance. In contrast, tactical decisions, such as developing a ‘Master Surgical Schedule,’ occur months in advance and are typically led by managerial staff. Operational decisions are short-term or same-day adjustments typically made by clinicians, often with input from multiple disciplines.^5 6^ However, the dynamic nature of surgical care, impacted by urgent cases, cancellations and resource fluctuations, makes aligning demand and capacity challenging.^7 8^ Since preoperative scheduling affects all critical resources across the perioperative pathway, including ward capacity, bed availability and intensive care, optimising these processes is essential to prevent delays in care, improve patient outcomes and reduce strain on other parts of the healthcare system.

The digitalisation of healthcare data and the adoption of electronic health records present promising opportunities to address the complexities of surgical scheduling and enhance clinical decision-making at all stages of the theatre management process. While previous literature has explored theatre planning,^68^,^13^ it has not examined how digital systems can address this complex problem. Similarly, prior systematic reviews have investigated the role of machine learning and artificial intelligence in perioperative medicine.^14 15^ However, these have not focused on preoperative scheduling or the practical, real-world application of such algorithms. Additionally, there is a limited understanding of which types of digital systems, such as dashboards or digital lists, are currently used for surgery booking and clinical decision-making.

As secondary care providers aim to achieve significant efficiency gains while delivering a safe and effective service to patients, they must have access to the best available evidence on which to base decisions regarding the use of new technology. This systematic review aims to address these gaps by examining the impact of digital surgery scheduling systems on the quality of preoperative care. Specifically, it will assess outcomes related to patient-centredness, safety, effectiveness, efficiency, timeliness of care and equity.

## Methods and analysis

A systematic literature review will follow the Cochrane Handbook for Systematic Reviews and use the Preferred Reporting Items for Systematic Reviews and Meta-Analysis (PRISMA) guidelines.^16 17^ This systematic review protocol adhered to the PRISMA-P reporting guidelines.^18^ This protocol has also been published in PROSPERO (CRD42024625469). The literature search and data analysis are scheduled to commence in July 2025 and are expected to conclude by September 2025.

### Search strategy

Identifying eligible studies will involve a comprehensive search (inception to present) across Ovid MEDLINE, Ovid EMBASE, HMIC and PsycINFO. No language limitations or time frame restrictions will be applied to ensure that all relevant literature is captured. The automation tool ‘Polyglot Search Translator’ was employed in the search strategy design, and keywords were guided by the keyword co-occurrence analysis performed by Rahimi and Gandomi.^6^ A specialist librarian with experience in evidence synthesis was involved in developing the search strategy (online supplemental appendix A).

### Eligibility criteria

The search strategy will be based on predefined eligibility criteria, as highlighted in table 1.

**Table 1:** Eligibility criteria as structured by PICOS

	Inclusion	Exclusion
Population	Individuals receiving preoperative care in preparation for an elective surgical procedure.	Individuals receiving perioperative care for an emergency procedure.
Intervention	A digital system for surgery scheduling (eg, EHR scheduling tools, dashboards, artificial intelligence-based optimisation systems) and management.	A digital system used solely for anaesthesia management and digital systems used during the intraoperative and postoperative phases.
Comparator	The usual or standard method for surgery scheduling (eg, paper-based scheduling, in-person or phone coordination, whiteboard tracking or communication via email or fax) without the use of technology.	No usual or standard method comparison.
Outcomes	Quantitative outcomes pertaining to the NAM Quality of Care Framework components: Patient-centredness (eg, satisfaction scores). Efficiency (eg, cost per procedure, staff time savings). Safety (eg, errors, mortality). Effectiveness (eg, clinical outcomes, completion rates for required tests). Timeliness (eg, time to surgery, delays). Equity (eg, variations by socioeconomic status, ethnicity and geographic location).	Qualitative outcomes and outcomes not related to the NAM domains of quality of care.
Study design	Randomised controlled trials, cluster randomised trials, quasi-experimental, case-control, cohort studies, cross-sectional and cost-effectiveness.	Commentaries, conference papers, case reports and reviews.

EHR, Electronic Health Record; NAM, National Academy of Medicine.

#### Population

This systematic review will focus on individuals receiving preoperative care in preparation for an elective surgical procedure. For this search, there will be no age restriction. Preoperative care will be defined as the period between the decision to undergo surgery and the day of surgery.^19^ An elective surgical procedure is planned rather than required to address an immediate medical emergency.^20^

#### Intervention

The intervention of interest is the use of a digital surgery scheduling system. This system can be defined as a digital system (eg, information management system, platform) that displays and/or processes data (dashboards, graphics, digital lists) for elective surgery scheduling and management during the preoperative phase of the perioperative period, including patient waitlist management. These systems may include administrative tools (eg, electronic health record-integrated scheduling modules, automated booking systems, centralised dashboards) and decision-support or predictive tools (eg, artificial intelligence-based scheduling systems). Hybrid systems combining both functions will also be included. There will be no restrictions on the discipline of those using this system, and eligible trials could assess a clinician using these systems with interdisciplinary input or in the context of a multidisciplinary team.

Surgery scheduling approaches, tools and techniques without a digital system will be excluded from this review. Digital systems used solely for anaesthesia management (ie, anaesthesia information management systems) and digital systems used for the perioperative intraoperative and postoperative phases will also be excluded.

#### Comparator

The comparator will be the standard method for scheduling surgery, without the use of digital systems or technology, for adults undergoing elective surgery. Examples include paper-based scheduling, phone or in-person coordination, informal scheduling methods and basic communication tools such as email or fax.

#### Outcomes

Primary outcomes will include measures related to the National Academy of Medicine (NAM) quality of care domains.^21^ For example, patient-centredness will be assessed through patient and staff satisfaction and engagement. Efficiency will be measured by cost-effectiveness, resource utilisation and healthcare expenditure. Safety will focus on reductions in scheduling errors, patient harm and complications resulting from inadequate preparation. Effectiveness will be examined through clinical outcomes, including adherence to preoperative protocols and surgical readiness. Timeliness will be evaluated based on changes in wait times, including the time to surgery and delays in preoperative assessments. Equity will assess disparities in access based on sociodemographic factors (age and sex), ethnicity and geography. We will also examine workflow improvements (eg, ease of use for staff, reduction in administrative workload) and provider perspectives (eg, surgeon/nurse satisfaction with digital tools).

#### Study design

Relevant studies with experimental and observational designs that provide quantitative data will be considered for inclusion in this review. Mixed-methods studies with a substantive quantitative component will also be considered for inclusion. However, qualitative designs, case reports, systematic reviews, meta-analyses, editorials, commentaries, letters, opinion pieces and unpublished studies will be excluded.

### Selection process

All eligible articles from the initial search will be imported into Covidence, where duplicates will be removed.^22^ Two reviewers, EL-E and GK, will independently perform the title, abstract and full-text screening according to the eligibility criteria. Reviewers will resolve any conflicts that arise through discussion. If necessary, a third reviewer will be consulted to resolve any disagreements that cannot be resolved through discussion.

### Data extraction

EL-E and GK will then extract the following data from the eligible studies in an Microsoft Excel spreadsheet: publication date, first author, study title, study design, time frame, sample size, participant group characteristics, comparator group characteristics, description of the digital surgery scheduling system, analysis methods and outcome measures.

### Quality assessment

Depending on the study design, a National Heart, Lung and Blood Institute quality assessment tool will be employed by EL-E for the Risk of Bias Quality assessment.^23^ GK will then verify this assessment.

### Data synthesis

A narrative synthesis approach will be used, collating the outcomes reported by the NAM quality-of-care domain and their measures of effect using tables in Microsoft Excel.^21^ Additionally, the descriptions of the intervention will be consolidated into tables or figures to identify common trends and themes using an appropriate taxonomy to define the interventions. If suitable, a meta-analysis of the extracted data will be conducted to quantify the impact of the digital surgery scheduling system on the reported outcomes.

### Ethics and dissemination

This systematic review will not require ethical approval as it aims to collate and summarise peer-reviewed, published evidence. It will be disseminated in peer-reviewed journals, conferences and patient-led lay summaries.

### Patient and public involvement

Although patients and the public were not directly involved in designing this systematic review protocol, we aim to engage patient partners through the Northwest London Applied Research Collaboration (National Institute for Health and Care Research). We are considering how patient partners could contribute to interpreting the results, co-developing a dissemination strategy and summarising the review for lay audiences.

## Supplementary material

10.1136/bmjopen-2025-102034 online supplemental file 1
