# Supplementary material for: Impact of digital surgery scheduling systems on the quality of preoperative care: a systematic review protocol
Source: BMJ Open. 2025 Jul 16;15(7):e102034. doi: 10.1136/bmjopen-2025-102034 (PMC12273116; doi:10.1136/bmjopen-2025-102034)
Supplement: online supplemental file 1 [file bmjopen-15-7-s001.docx]

**Supplementary Materials 1**

**Table 1**: Search strategy (OVID Embase).

| 1. *Search items* | *Concepts* |
| --- | --- |
| 1. ((surgery* or operation* or "surgical procedure*" or "surgical care" or "surgical management" or "operating room" or "operating theatre") and ((schedul* or plann* or coordinat* or timetable* or book* or logistic* or calendar* or appointment* or workflow* or "queue management" or "resource allocation") adj3 (system* or platform* or software or tool* or interface* or dashboard* or "digital solution" or "decision support system"))) | Digital surgery scheduling system |
| 1. (((experience* or satisfaction) adj4 (patient* or consumer* or client* or survey* or questionnaire*)) or PREM* or patient-reported experience measure* or patient-cent?red* or person-cent?red*) 2. Patient Satisfaction/ 3. 2 or 3 | Patient-centredness |
| 1. Treatment Outcome/ or ((health or clinical* or treatment*) adj3 (outcome* or effective* or efficacy)) | Effectiveness |
| 1. ((patient adj3 (safety or harm)) or misdiagnos* or safety manag* or (accident* adj2 prevent*) or error* or medication reconcil* or near miss*) 2. patient harm/ or patient safety/ or Diagnostic Errors/ 3. 6 or 7 | Safety |
| 1. (efficiency or economic* or cost* or expenditure* or charge* or (number adj3 appointment*) or (number adj3 admission*) or (number adj3 consultation*)) | Efficiency |
| 1. (wait* list* or wait* time* or timeliness) 2. Time-to-Treatment/ or Waiting Lists/ 3. 10 or 11 | Timeliness |
| 1. ((health* or health care or access) adj3 (equity or disparit* or inequit* or inequalit* or equality or gap)) 2. Health Equity/ 3. 13 or 14 | Equity |
| 1. 4 or 5 or 8 or 9 or 12 or 15 2. 1 and 16 |  |
