# Supplementary material for: Impact of digital surgery scheduling systems on the quality of preoperative care: a systematic review protocol
Source: BMJ Open. 2025 Jul 16;15(7):e102034. doi: 10.1136/bmjopen-2025-102034 (PMC12273116; doi:10.1136/bmjopen-2025-102034)
Supplement: Supplementary file 2 [file bmjopen-2025-102034.reviewer_comments.pdf]

PEER REVIEW HISTORY

BMJ Open publishes all reviews undertaken for accepted manuscripts. Reviewers are asked to complete a checklist review form and are provided with free text boxes to elaborate on their assessment. These free text comments are reproduced below.

ARTICLE DETAILS

Title (Provisional)

The impact of digital surgery scheduling systems on the quality of pre-operative care: a systematic review protocol

Authors

Lammila-Escalera, Elena; Kerr, Gabriele; Greenfield, Geva; Hayhoe, Benedict; Brewer, Natalie; Antonacci, Grazia; Majeed, Azeem; Neves, Ana Luisa

---

VERSION 1 - REVIEW

**Reviewer**	**1**
**Name**	**Harrison, Ewen**
**Affiliation**	**University of Edinburgh**
**Date**	**09-May-2025**
**COI**	**None**

Thank you for the opportunity to review "The impact of digital surgery scheduling systems on the quality of pre-operative care: a systematic review protocol".

It looks good and the study will be interesting.

A few considerations:

1. The comparator definition is vague ("usual or standard method") and will vary across studies. Standardising what is considered "usual care" (e.g., manual, paper-based scheduling or non-digital methods) may improve consistency.

2. Similarly, digital surgery scheduling systems vary widely (dashboards, automated lists, EHR-integrated systems, predictive scheduling tools). Clearly defining these upfront (e.g., predictive algorithms vs administrative tools) may help manage heterogeneity in analysis.

3. Finally, I don't feel strongly about the 2010 cutoff, but I first saw such a system being used in 1997, so make sure you are not missing early formative work.

**Reviewer**	**2**
**Name**	**Akyürek, Çağdaş Erkan**

**Affiliation**	**Ankara University, Health Management**
**Date**	**16-Jun-2025**
**COI**	**None**

Thank you for your valuable contribution to the field. It will be exciting to read the complete article after publication.

VERSION 1 - AUTHOR RESPONSE

**Reviewer Comments**	**Author Response**
**REVIEWER #1**:	
1. The comparator definition is vague ("usual or standard method") and will vary across studies. Standardising what is considered "usual care" (e.g., manual, paper-based scheduling or non-digital methods) may improve consistency.	Thank you for this feedback. To address this, we have revised the "Comparator" section of the "Methods" to include a more specific description:<br>"*Examples include paper-based scheduling, phone or in-person coordination, informal scheduling methods and basic communication tools such as email or fax*."<br><br>For consistency, we have also updated Table 1 (comparator row, inclusion column) to reflect these examples:<br>"*e.g. paper-based scheduling, in-person or phone coordination, whiteboard tracking, or communication via email or fax*".
2. Similarly, digital surgery scheduling systems vary widely (dashboards, automated lists, EHR-integrated systems, predictive scheduling tools). Clearly defining these upfront (e.g., predictive algorithms vs administrative tools) may help manage heterogeneity in analysis.	We have now expanded the description in the "Intervention" section of the "Methods" to clarify the type of systems eligible for inclusion in the review: *"These systems may include administrative tools (e.g. EHR-integrated scheduling modules, automated booking systems, centralised dashboards) and decision-support or predictive tools (e.g. artificial intelligence-based scheduling systems). Hybrid systems combining both functions will also be included."*<br><br>Additionally, we have updated Table 1 (intervention row, inclusion column) to reflect the new definition: *A digital system for surgery scheduling (e.g. EHR scheduling tools, dashboards, artificial intelligence-based optimisation systems) and management.*
3. Finally, I don't feel strongly about the 2010 cutoff, but I first saw such a system being used in 1997, so make sure you are not missing early formative work.	Thank you for your helpful comment - we agree. Consequently, we have revised the Search Strategy section in the Methods, changing the search cut-off date from 2010 to inception of database, to be able to capture all relevant literature that may have been missed with the previous cut-off. Specifically, we now state, "*Identifying eligible studies will involve a comprehensive search (inception to present) across Ovid MEDLINE, Ovid EMBASE, HMIC, and PsycINFO. No language limitations or timeframe restrictions will be applied to ensure that all relevant literature is captured.*"

	We have also updated the abstract and PROSPERO record to reflect this change.
